# Amyloid Precursor Protein Is Required for Normal Function of the Rod and Cone Pathways in the Mouse Retina

**DOI:** 10.1371/journal.pone.0029892

**Published:** 2012-01-18

**Authors:** Tracy Ho, Kirstan A. Vessey, Roberto Cappai, Virginie Dinet, Frédéric Mascarelli, Giuseppe D. Ciccotosto, Erica L. Fletcher

**Affiliations:** 1 Department of Anatomy & Cell Biology, The University of Melbourne, Melbourne, Victoria, Australia; 2 Department of Pathology, The University of Melbourne, Melbourne, Victoria, Australia; 3 Bio21 Molecular Science and Biotechnology Institute, The University of Melbourne, Melbourne, Victoria, Australia; 4 Centre de Recherche des Cordeliers, Université Paris Descartes, Paris, France; 5 Institut National de la Santé et de la Recherche Médicale, Paris, France; 6 Université Pierre et Marie Curie, Paris, France; Massachusetts Eye & Ear Infirmary, Harvard Medical School, United States of America

## Abstract

Amyloid precursor protein (APP) is a transmembrane glycoprotein frequently studied for its role in Alzheimer's disease. Our recent study in APP knockout (KO) mice identified an important role for APP in modulating normal neuronal development in the retina. However the role APP plays in the adult retina and whether it is required for vision is unknown. In this study we evaluated the role of APP in retinal function and morphology comparing adult wildtype (WT) and APP-KO mice. APP was expressed on neuronal cells of the inner retina, including horizontal, cone bipolar, amacrine and ganglion cells in WT mice. The function of the retina was assessed using the electroretinogram and although the rod photoreceptor responses were similar in APP-KO and WT mice, the post-photoreceptor, inner retinal responses of both the rod and cone pathways were reduced in APP-KO mice. These changes in inner retinal function did not translate to a substantial change in visual acuity as assessed using the optokinetic response or to changes in the gross cellular structure of the retina. These findings indicate that APP is not required for basic visual function, but that it is involved in modulating inner retinal circuitry.

## Introduction

Amyloid precursor protein (APP) is a type I transmembrane glycoprotein that is endogenously expressed in the brain and retina [1]. APP is well known for its role in pathology, as its cleavage product Aβ peptide is the key component of amyloid plaques in Alzheimer's disease [2], [3]. However, the normal function of APP in the central nervous system remains to be clarified. APP transgenic and APP knockout (KO) mouse models indicate that APP has physiological functions involving modulation of neurite outgrowth [4], synaptic activity [5], [6], [7], regulation of metal homeostasis [8], [9], [10], synaptic transmission [11] and synaptic adhesion at the neuromuscular junction [12].

We have reported that APP plays a role in retinogenesis, as APP-KO mice have changes in retinal cell development. In particular, differentiation of some inner retinal neurons, specifically horizontal and amacrine cells are hampered in APP-KO mice during early postnatal development [13]. By adulthood APP-KO mice have normal numbers of horizontal cells and most types of amacrine cells. However, the number of AII amacrine cells, which are important for rod pathway function, are still reduced in the adult APP-KO retina [13]. We hypothesised that these alterations in retinal cell development may affect signalling in the adult APP-KO mouse. Therefore, we investigated the role of APP in the retina by assessing the function of the rod and cone pathways separately using the electroretinogram (ERG) and general visual function using the optokinetic response. We further characterised the cellular location of APP in the adult retina and the effect of APP deficiency on retinal morphology.

## Methods

### Animals

Mice were housed in the Biomedical Sciences Animal Facility at The University of Melbourne. Adult APP-KO mice [14] and age-matched wild type (WT) C57/Bl6J×SV129 control mice at 1, 3, and 12 months of age were used. Mice were housed with *ad libitum* access to food and water under a 12 h∶12 h light-dark cycle. All experiments involving animals were approved by the University of Melbourne animal experimentation ethics committee, Ethics ID numbers: 0911158 & 1011059. In addition, experiments adhered to the ARVO Statement for the Use of Animals in Ophthalmic and Vision Research. For *in vivo* experiments, mice were anaesthetised by intra-peritoneal administration of a mixture of ketamine (67 mg/kg) and xylazine (13 mg/kg). For all procedures involving tissue collection, mice were first anesthetised (as above) and euthanized by cervical dislocation.

### Immunohistochemistry

Immunohistochemistry was used to determine the neuron specific location of APP in normal retina and to determine whether retinal morphology was altered in the APP-KO using previously published techniques [15]. Briefly posterior eyecups were fixed for 30 minutes in 4% paraformaldehyde, washed in 0.1 M phosphate buffer, pH 7.4 (PB) and then cryoprotected in graded sucrose solutions. Eyes were embedded in OCT compound (Tissue-Tek, California, USA) and transverse sections of the eyecup were cut with a Microm HM550 cryostat (Microm, Walldorf, Germany) at −20°C, at a section thickness of 14 µm. Sections were mounted onto Polysine® glass slides (Polysine® Adhesion slides, Thermo Scientific, VIC, Australia) and stored at −20°C until processing.

For staining, thawed sections were washed in PB and non-specific binding sites were blocked (10% Normal goat serum (NGS), 1% Bovine serum albumin, 0.5% Triton X-100, in PB) for 1 hour. The sections were incubated with primary antibodies overnight (Table 1). After washing in PB, sections were incubated with secondary antibody: goat anti-guinea pig, goat anti-mouse, goat anti-rabbit or donkey anti-sheep conjugated to AlexaFluor 488, AlexaFluor 594 or AlexaFluor 643 (Invitrogen, VIC, Australia; diluted 1∶500) as required, for 90 minutes. The sections were washed and cover slipped. Two different antibodies were used to localise APP expression in the adult mouse retina; CT20, (rabbit anti-APP; Calbiochem, Merck Australia), which was generated against the C-terminal region of the APP peptide and 22C11, (mouse anti-APP; Millipore, Invitrogen Australia), which was generated against the N-terminal region of the APP peptide. Preliminary studies with these antibodies indicated that while the CT20 antibody produced specific labelling in WT but not APP-KO mouse retina, the 22C11 antibody labelled similarly in both. In particular, 22C11 labelled retinal glia, the Müller cells, in both WT and APP-KO mouse retina. This indicates that the antibody, 22C11, generated against the N-terminal epitope of APP could not be used to specifically evaluate APP expression in the retina, possibly because it binds another protein with a similar N-terminal, APLP2, and not just APP [16]. However, as the CT20 antibody was specific for APP in the retina all further experiments were completed using this antibody. For immunohistochemical localisation of APP on retinal ganglion cells, Thy1-HYFP mice, which have subpopulations of ganglion cells that produce yellow fluorescent protein in cells which express the thy-1 promoter, were used [17]. These mice were kindly donated by A/Prof Anthony Hannan (Howard Florey Institute, Vic, Australia) and bred at the University of Melbourne.

**Table 1:** Primary antibodies used for immunohistochemical analysis.

Cell type/Target	Antibody	Dilution	Source	Reference
APP	Rabbit anti-APP CT20	1∶1000	Calbiochem (Merck), VIC, Australia	[56], [57]
APP and N-terminal	Mouse anti-APP 22C11	1∶100	Millipore, North Ryde, NSW, Australia	[13], [58]
Horizontal cells	Mouse anti-Calbindin D28k	1∶4000	Swant, Bellinzona, Switzerland	[59]
Rod Bipolar cells	Mouse anti-PKCα	1∶400	Sigma-Aldrich, NSW, Australia	[60]
Cone-ON& OFF Bipolar cells	Mouse anti-ZNP-1	1∶2000	ZIRC, Eugene, Oregon, USA	[61]
Amacrine cells	Mouse anti-Calretinin	1∶1000	Swant, Bellinzona, Switzerland	[62]
GABAergic amacrine cells	Guinea pig anti-GABA	1∶500	Millipore, NSW, Australia	[62]
Ganglion and GABAergic amacrine cells	Mouse anti-NeuN	1∶500	Millipore, NSW, Australia	[63]
Ganglion cells	Goat anti-GFP (for YFP under Thy1 promoter)	1∶500	Rockland, SA, Australia	[17]

Images were taken using the LSM 5 Pascal confocal laser scanning microscope (Zeiss, Oberkochen, Germany) using a 40x/1.3 oil-immersion objective at a resolution of 1024×1024 pixels. Gain settings were at the same level when taking images for WT and APP-KO tissue sections. Magnifications varied, with scale bars digitally added to the images by Zeiss LSM Image Browser software (v4.2.0.121, Zeiss, Oberkochen, Germany). Images from WT and APP-KO mice were adjusted for black levels, contrast and brightness in Adobe Photoshop CSE version 4 (Adobe Systems, CA, USA) using the same settings for consistency between samples.

### Visual function of WT and APP-KO mice

**Electroretinography (ERG) and component analysis:** The ERG was used to assess the function of the rod and cone pathways of 3 month old WT and APP-KO mice using previously published techniques [15]. Mice were dark adapted overnight and anesthetised under dim red light. Pupils were dilated with tropicamide (Mydriacyl 0.5%, Alcon Laboratories, Austral ia) and the cornea was anaesthetised with proxymetacaine (Alcaine 0.5%, Alcon Laboratories, Australia). ERG responses were recorded with custom-made silver-silver chloride (Ag-AgCl) electrodes, with a corneal active electrode that measured the summed response with reference to an inactive electrode hooked onto the front teeth.

ERGs were elicited with full-field flashes of 2.1 log cd.s/m^2^ intensity (Nikon SB900, NSW, Australia), presented with a custom Ganzfeld sphere. The rod and cone responses were isolated using a twin-flash paradigm with a 0.8 s interstimulus interval [18], [19]. Responses were amplified (gain×5000; −3 dB at 1 Hz and 1 kHz, ADInstruments, NSW, Australia) and digitized at 10 kHz. Stimuli and ERG recordings were coordinated using Scope v3.6.9 program software.

**Rod response:** The rod photoreceptor response of the ERG is represented by the cornea negative a-wave. It was fitted to a modified version of the PIII model [20]:
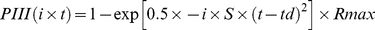
where PIII (µV) is the summed rod photocurrent as a function of luminous energy, i (cd.s.m^−2^) and time, t (seconds). Sensitivity, S (m^2^cd^−1^s^−3^) represents the gain of phototransduction cascade with a time delay, t_d_ (seconds) that accounts for the brief latency between stimulus presentation and recording of response.

The positive b-wave reflects post-photoreceptoral function and was isolated by subtraction of the rod PIII from the raw rod waveform and then fitted using an inverted gamma function to generate the rod PII [21]:
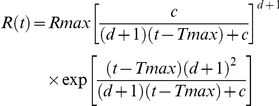
From the rod PII the response amplitude Rmax (µV) and time to peak, t (milliseconds (ms)) after the flash onset were calculated. The parameters for the equation include: the peak amplitude, Rmax (µV), the implicit time until the maximum amplitude, Tmax (ms), and constants, *c* and *d*, are factors that affect the shape of the curve.

The Oscillatory potentials (OPs) are thought to primarily reflect the excitatory and inhibitory activities of the amacrine cells [22]. They were isolated from the ascending limb of the b-wave by subtracting the fitted rod PII from the raw rod waveform. OP2, OP3 and OP4 were analysed to assess their amplitude (µV) and their implicit time (ms). OP1 and OP5 were not assessed as they are difficult to consistently distinguish from noise due to their small amplitude.

**Cone response:** The cone a-wave of the mouse is too small to be analysed, due to the small number of cone photoreceptors as compared to the rod photoreceptors. Nevertheless, the cone post-receptoral response (cone b-wave) could be assessed and the cone PII was analysed by fitting the inverted gamma function (above) to the raw cone waveform. The maximum amplitude response, Cone PII amplitude (µV), and the time when this was reached, implicit time (ms) were assessed.

**In vivo, visual spatial threshold measurements:** Visual function was assessed in live, behaving animals using a virtual optomotor system (OptoMotry 1.7.4.3 [23]). The optokinetic response of WT and APP-KO mice under light adapted conditions was determined in response to sine wave gratings presented at 100% contrast. Spatial frequency thresholds were determined using a simple staircase protocol that varied the spatial frequency of gratings rotating at a constant velocity (12 deg s^−1^). Using a two-alternative, forced-choice protocol, the observer would choose the direction of grating rotation (right or left) according to the mouse's head movements. The average spatial frequency cutoff (cycles/degree) was defined as the highest spatial frequency that elicited a reliable head tracking response. Mice were tested at a photopic mean luminance (100 cd m^−2^). All measurements were made by an observer blind to stimulus condition.

### Statistical analysis

Results are expressed as the mean ± the standard error of the mean (SEM). Spatial frequency threshold across ages (1, 3 and 12 months) and genotype (WT vs. APP-KO) were analysed by two-way, analysis of variance (2-way ANOVA), and a Bonferroni post-hoc test was used to analyse the effect of genotype at individual ages (Graphpad Prism v.4, San Diego, CA, USA). ERG responses between WT and APP-KO mice at 3 months were compared using an unpaired Student's t-test. In all figures, statistical significance is expressed as *P<0.05, **P<0.01, and ***P<0.001.

## Results

### APP is expressed in the inner neural retina of the WT mouse

**Figure 1:**
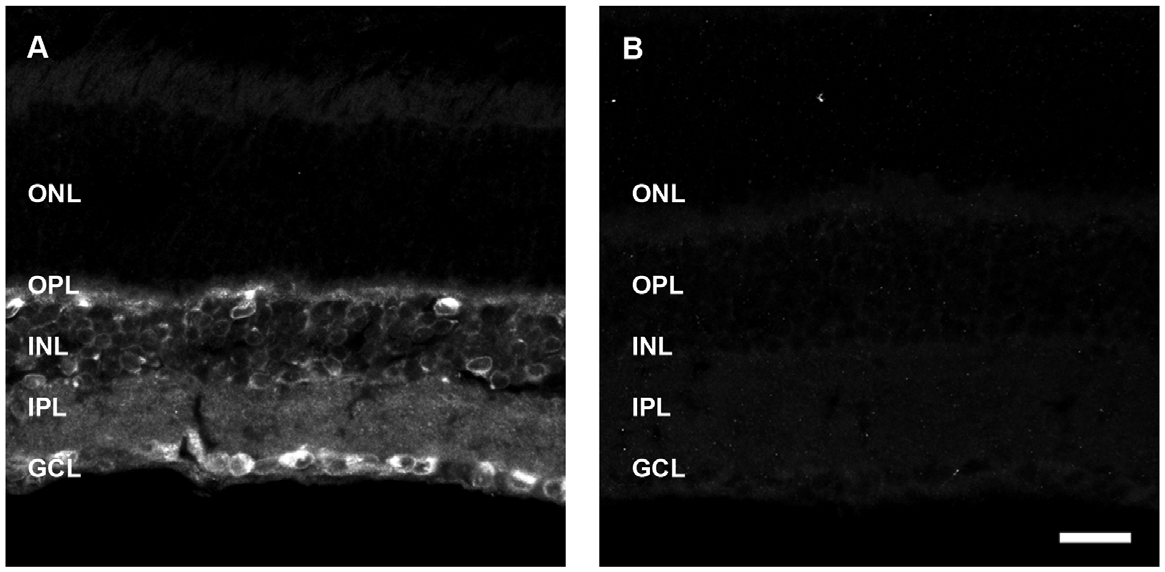
The antibody CT20 detects APP-immunoreactive protein in WT but not APP-KO retina. (A) Immunohistochemistry on a transverse retinal section from an adult WT mouse shows CT20 labels APP immunoreactivity in the outer plexiform layer (OPL), inner nuclear layer (INL), inner plexiform layer (IPL) and ganglion cell layer (GCL) but not in the outer nuclear layer (ONL). (B) The APP-KO retina does not label with the CT20 antibody, suggesting that the antibody is specific for APP in retinal sections. Scale, 20 microns.

The APP antibody, CT20 binds to the intracellular C-terminal domain of APP and we confirmed its specificity for APP in transverse sections of neural retina from three month old WT and APP-KO mice. In WT neural retina, CT20 labelled cells of the inner nuclear layer (INL) and ganglion cell layer (GCL) but not photoreceptor nuclei in the outer nuclear layer (ONL; Figure 1A). In contrast, in APP-KO mice, there was no specific labelling detected in the retina in the absence of APP (Figure 1B). This finding indicates that the CT20 antibody can be used to specifically detect APP expression in the retina by immunohistochemistry.

**Figure 2:**
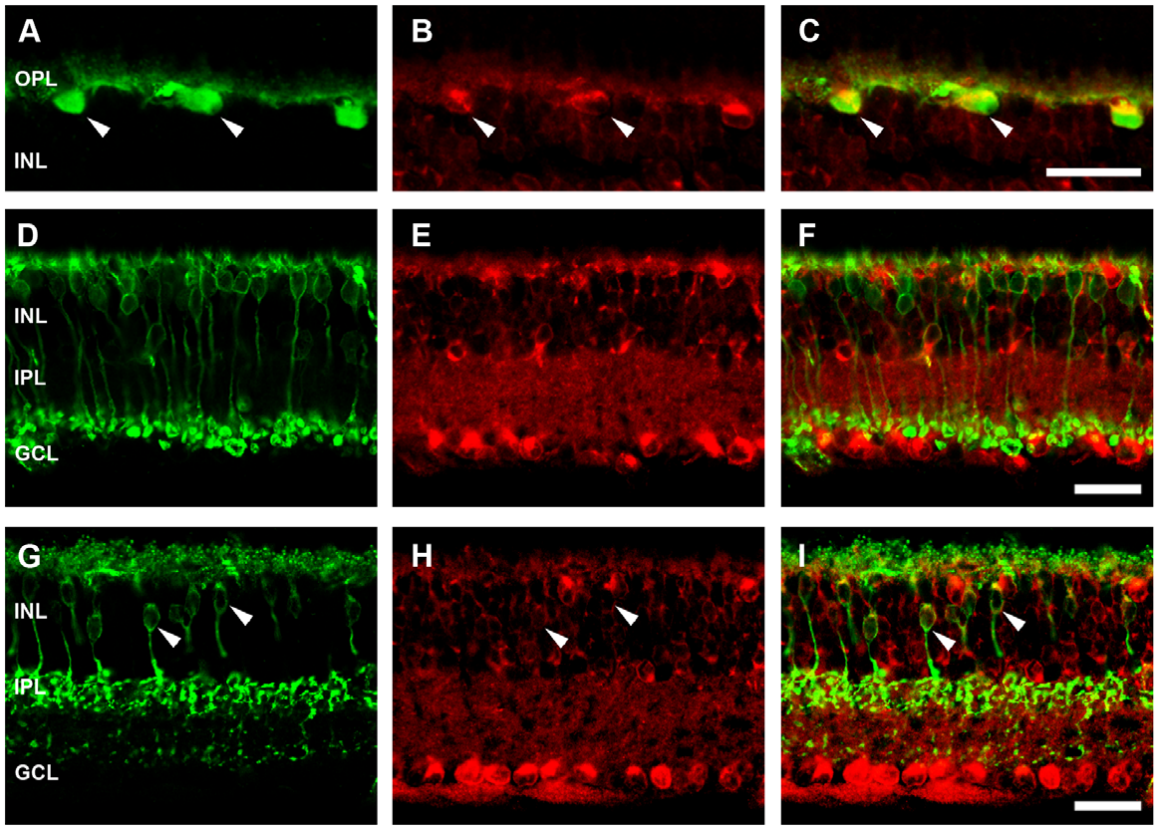
APP-immunoreactivity (IR) in the WT mouse retina colocalises with horizontal cells and cone bipolar cells but not rod bipolar cells. Immunohistochemistry on a transverse retinal section from an adult WT mouse for (A) horizontal cells labelled with Calbindin, and (B) APP-IR, showing (C) colocalisation. Immunohistochemistry for (D) rod bipolar cells labelled with PKCα and (E) APP-IR, showing that the markers do not colocalise (F). Immunohistochemistry for (G) cone bipolar cells (Type 2 and 6) labelled with a ZNP-1 and (H) APP-IR, showing (I) colocalisation. Arrows indicate examples of colocalisation. Outer plexiform layer (OPL), inner nuclear layer (INL), inner plexiform layer (IPL) and ganglion cell layer (GCL). Scale, 50 microns.

**Figure 3:**
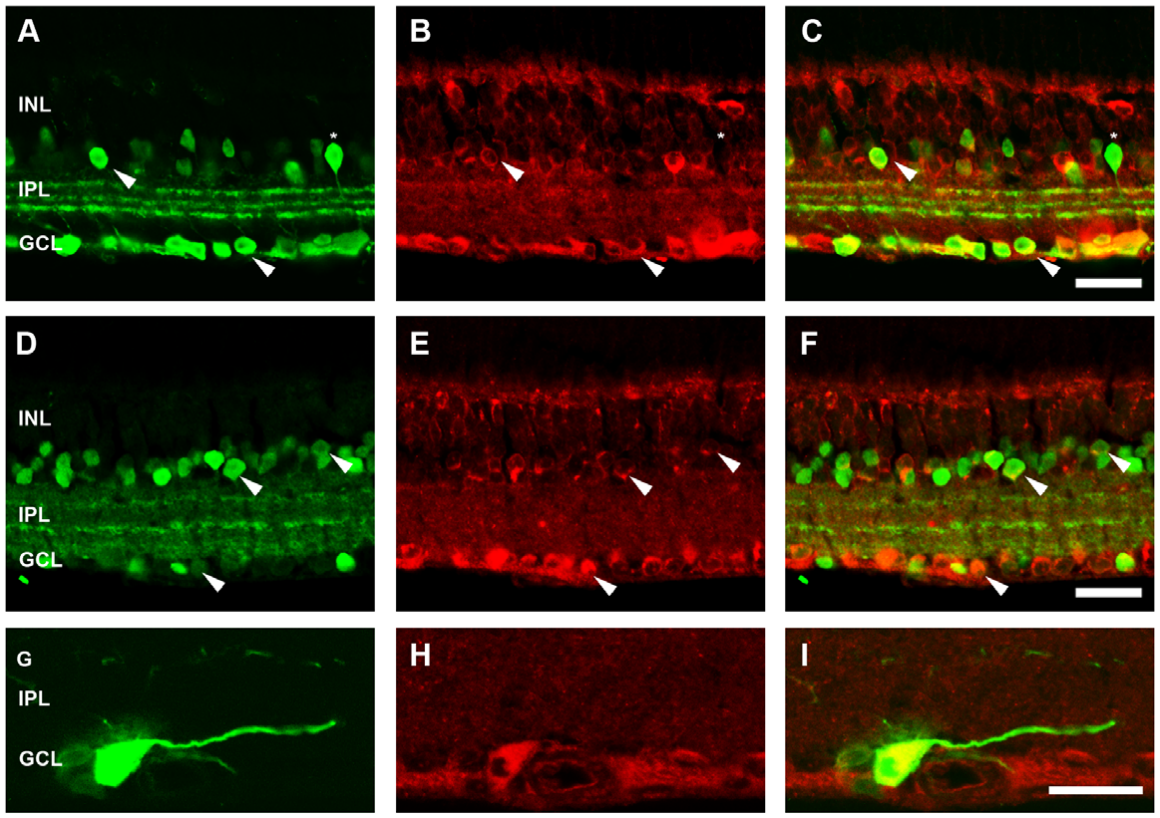
APP-immunoreactivity (IR) in the WT mouse retina colocalises with with calretinin positive- and GABA positive-amacrine cells, as well as ganglion cells. Immunohistochemistry on a transverse retinal section from an adult WT mouse for (A) amacrine cells labelled with Calretinin, and (B) APP-IR, showing (C) colocalisation. Immunohistochemistry for (D) GABAergic amacrine labelled with an antibody against GABA and (E) APP-IR, showing (F) colocalisation. Immunohistochemistry for (G) ganglion cells labelled with an antibody against GFP to localise YFP positive cells in the thy1-HYFP mouse and (H) APP-IR, showing (I) colocalisation. Arrows indicate examples of colocalisation, while * indicates a calretinin positive cell not labelled for APP-IR. Inner nuclear layer (INL), inner plexiform layer (IPL) and ganglion cell layer (GCL). Scale, 50 microns.

To determine the retinal cell types that express APP in the adult WT mouse, we performed double labelling experiments with CT20 and known retinal cell markers. Given the lack of CT20 labelling in the outer nuclear layer, we restricted our cellular analysis to the inner retinal neurons. Horizontal cells, labelled using an antibody against calbindin, were APP immunoreactive (Figure 2A–C), however, rod bipolar cells labelled with PKCα (Figure 2D–F) were not APP immunoreactive. Cone bipolar cells, specifically type 2-OFF and type 6-ON labelled with ZNP-1 (Figure 2G–I), co-localised with CT20 indicating that subpopulations of cone bipolar cells express APP. Amacrine cells, both calretinin-positive (Figure 3A–C) and GABA-positive (Figure 3D–F), were APP-immunoreactive, confirming our previous report of amacrine cell expression of APP [13]. To assess ganglion cells, we used a transgenic mouse where YFP expression is driven by the Thy-1 promoter in a subpopulation of ganglion cells (HYFP-thy1; [17]) and found CT20 co-localised with GFP positive ganglion cells (Figure 3G–I).

The co-localisation studies demonstrate that APP is expressed by virtually all neuronal cell types in the inner retina with the exception of rod bipolar cells in the adult mouse. Based on these findings, we predict that the lack of APP expression in APP-KO mice could affect inner retinal function.

### Rod pathway function is altered in the APP-KO mouse

**Figure 4:**
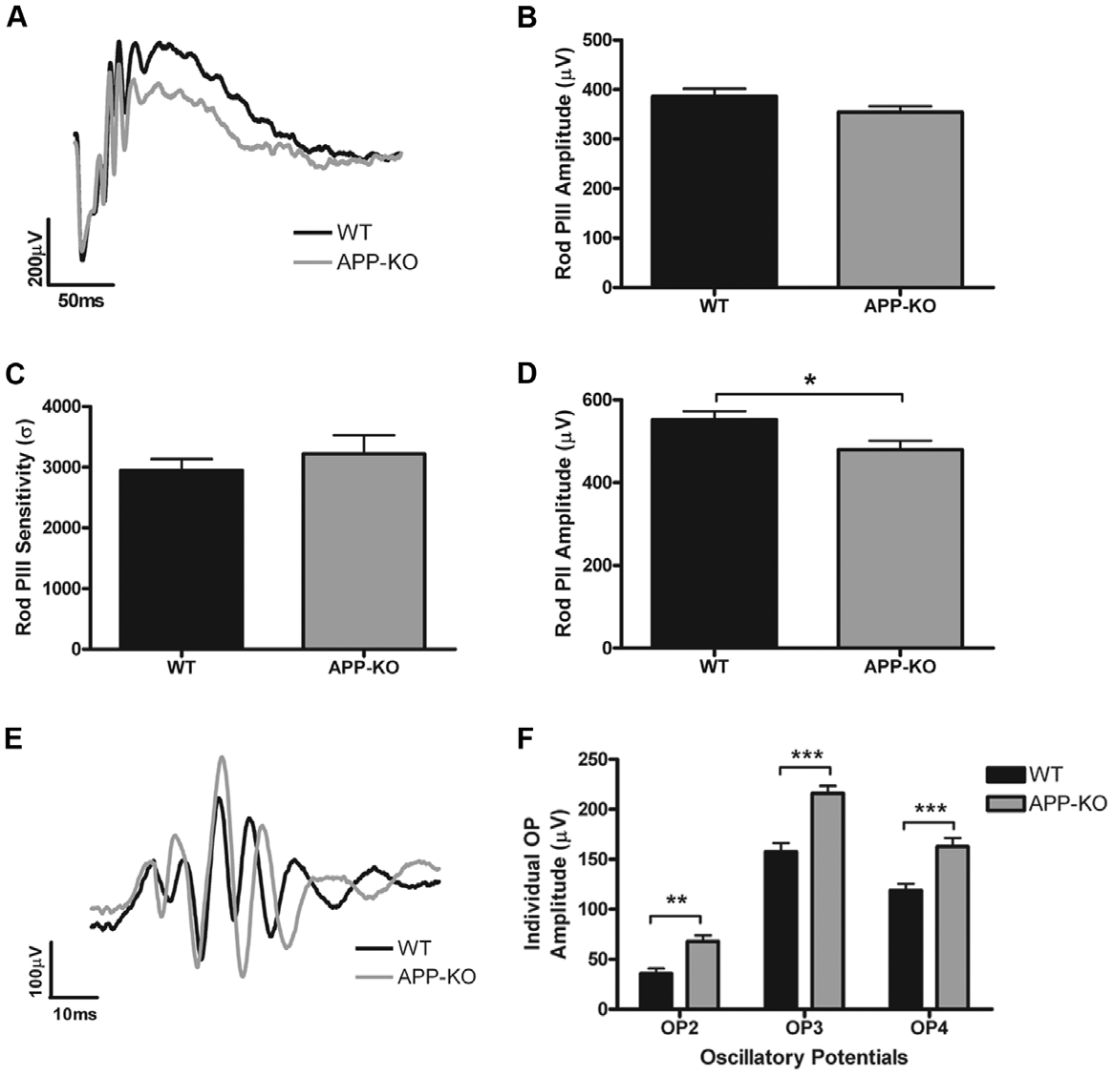
The function of the rod pathway is altered in the adult APP-KO mouse. (A) Representative waveforms of the rod ERG recorded from dark adapted, 3 month old WT (black) and APP-KO (grey) mice in response to a 2.1 log cd.s/m^2^ intensity flash. The photoreceptor derived, rod PIII was not altered in either (B) amplitude or (C) sensitivity in the APP-KO mouse when compared with the WT response. The post-photoreceptor response, the rod PII (D) was significantly smaller in amplitude in the APP-KO mouse. (E) Representative waveforms of the oscillatory potentials (OPs) extracted from rod ERG in WT (n = 16) and APP-KO (n = 14) mice. (F) All of the inner retinal derived OPs measured were significantly enhanced in the APP-KO mouse. Results expressed as mean +/− SEM, *, p<0.05, **, p<0.01; ***, p<0.001.

To assess the function of APP in the retina, the rod and cone ERGs from three month old WT and APP-KO mice were recorded. The rod and cone ERG waveforms represent a measure of the summed electrical responses of populations of retinal neurons in response to a flash of light. As such the ERG can be used as a measure of function of each of these neuronal populations. Figure 4A shows that the representative rod ERG waveform of APP-KO mice is altered when compared with the response recorded from WT mice. The rod photoreceptor response (PIII amplitude; a-wave; Figure 4B) of the APP-KO mice (354.40±12.14 µV, *n* = 14) was similar to that of the WT mice (386.10±15.75 µV, *n* = 16; *P* = 0.19). Moreover, the sensitivity parameter of the rod PIII (Figure 4C) that represents the kinetics of rod phototransduction, was not significantly different between the two genotypes (WT: 2948±186.10 m^2^cd^−1^s^−3^, *n* = 16; APP-KO: 3223±302.70 m^2^cd^−1^s^−3^, *n* = 14; *P* = 0.12). In contrast, the rod b-wave amplitude (rod PII), which represents inner retinal function primarily the rod bipolar cell response [24], [25], was significantly reduced in the APP-KO mice (480.0±21.11 µV, *n* = 14) compared to the WT mice (552.20±20.28 µV, *n* = 16; *P* = 0.02) (Figure 4D). However, the implicit time of this response was indistinguishable between APP-KO and WT mice (data not shown). The oscillatory potentials (OPs) extracted from the leading edge of the b-wave (Figure 4E) are thought to primarily reflect the functions of the amacrine cells [22], [26]. The average response amplitudes of the individual OPs were significantly greater in APP-KO mice (OP_2_: 35.73±5.04 µV; OP_3_: 215.90±7.69 µV; OP_4_: 162.90±8.40 µV; *n* = 14) than in WT mice (OP_2_: 67.88±5.88 µV, *P*<0.01; OP_3_: 157.30±9.09 µV, *P*<0.001; OP_4_: 118.90±6.65 µV, *P*<0.001; *n* = 16) (Figure 4F). However, there was no change in the timing of the OPs between these genotypes (data not shown). These data indicate APP is not important for rod photoreceptor function but that it is involved in modulating transmission of the inner retinal rod pathway response in adult mice.

### Cone pathway function is altered in the APP-KO mouse

**Figure 5:**
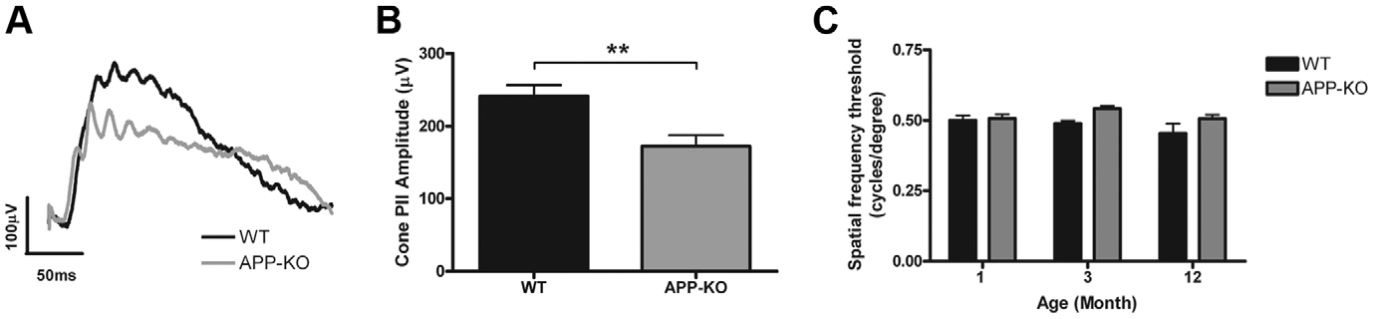
The function of the cone pathway is altered in the APP-KO mouse. (A) Representative waveforms of the cone ERG recorded from 3 month old WT (black) and APP-KO (grey) mice in response to the second flash of a twin flash paradigm ERG (2.1 log cd.s/m^2^ flash intensity). (B) The cone pathway post-photoreceptor response, the cone PII was found to be smaller in amplitude in APP-KO mice when compared with the WT response. (C) The photopic visual acuity, assessed using the optokinetic reflex to determine the spatial frequency threshold, was physiologically similar between APP-KO and WT mice with age. Results expressed as mean +/− SEM. **, p<0.01.

Representative cone ERG waveforms for APP-KO and WT mice, generated in response to the second flash of the twin-flash ERG, are presented in Figure 5A. The cone a-wave of the mouse is too small to be analysed, due to the small number of cone photoreceptors as compared to the rod photoreceptors. However, we could measure the cone b-wave, which has been proposed to reflect the activity of the cone depolarising bipolar cells in the INL [27], [28]. The cone b-wave amplitude (cone PII) was significantly smaller in the APP-KO mice (172.30±15.62 µV; *n* = 14) than in the WT mice (241.70±14.84 µV; n = 16) (Figure 5B). However, similar to the other kinetic parameters measured for the rod pathway, the cone PII implicit time was similar between the two genotypes (data not shown). These data suggest APP plays a role in cone pathway function in the mouse retina.

Given the prominent differences obtained from the ERG responses, we wanted to further identify whether APP-KO mice had functional deficits that may affect their visual acuity. This was assessed using the OptoMotry system, where the ability of the mice to track gratings of various widths was quantified by the optokinetic reflex and measured as the spatial frequency threshold, or visual acuity [29]. This test was performed under light adapted conditions; hence the response would primarily be due to functions of the cone pathway and in particular amacrine cells that are sensitive to changes in direction/lateral movement [30], [31], [32], [33]. The spatial frequency thresholds of WT and APP-KO mice were compared at one month (WT: 0.50±0.02 cyc/deg, *n* = 10; APP-KO: 0.51±0.02 cyc/deg, *n* = 8), three months (WT: 0.49±0.04 cyc/deg, *n* = 16; APP-KO: 0.54±0.03 cyc/deg, *n* = 14) and twelve months (WT: 0.45±0.04 cyc/deg, *n* = 13; APP-KO: 0.51±0.01, *n* = 10) of age (Figure 5C). A two-way ANOVA was performed to analyse the effects of age and genotype on spatial frequency threshold and although there was no significant effect of age (*P* = 0.162), there was a small but significant effect of genotype (*P* = 0.023). When a post-test analysis was used to compare the spatial frequency threshold of APP-KO and WT mice at each individual age, no significant differences were found at any specific age (Bonferroni post-test, 1 month *P>0.05*, 3 months *P>0.05* and 12 months *P>0.05*). As the spatial frequency thresholds of WT and APP-KO mice were at a physiological level statistically indistinguishable, this suggests that APP is not critical for function of this visual response.

Based on the significant differences in ERG b-waves, and OP amplitudes between WT and APP-KO mice, we conclude that APP has a functional role on the rod and cone pathways, where it is involved in modulating bipolar and amacrine cell functions in the inner retina. These functional findings are consistent with the cellular location of APP immunoreactivity in the retina.

### Inner retinal neuron profiles are similar in three month old WT and APP-KO mice

**Figure 6:**
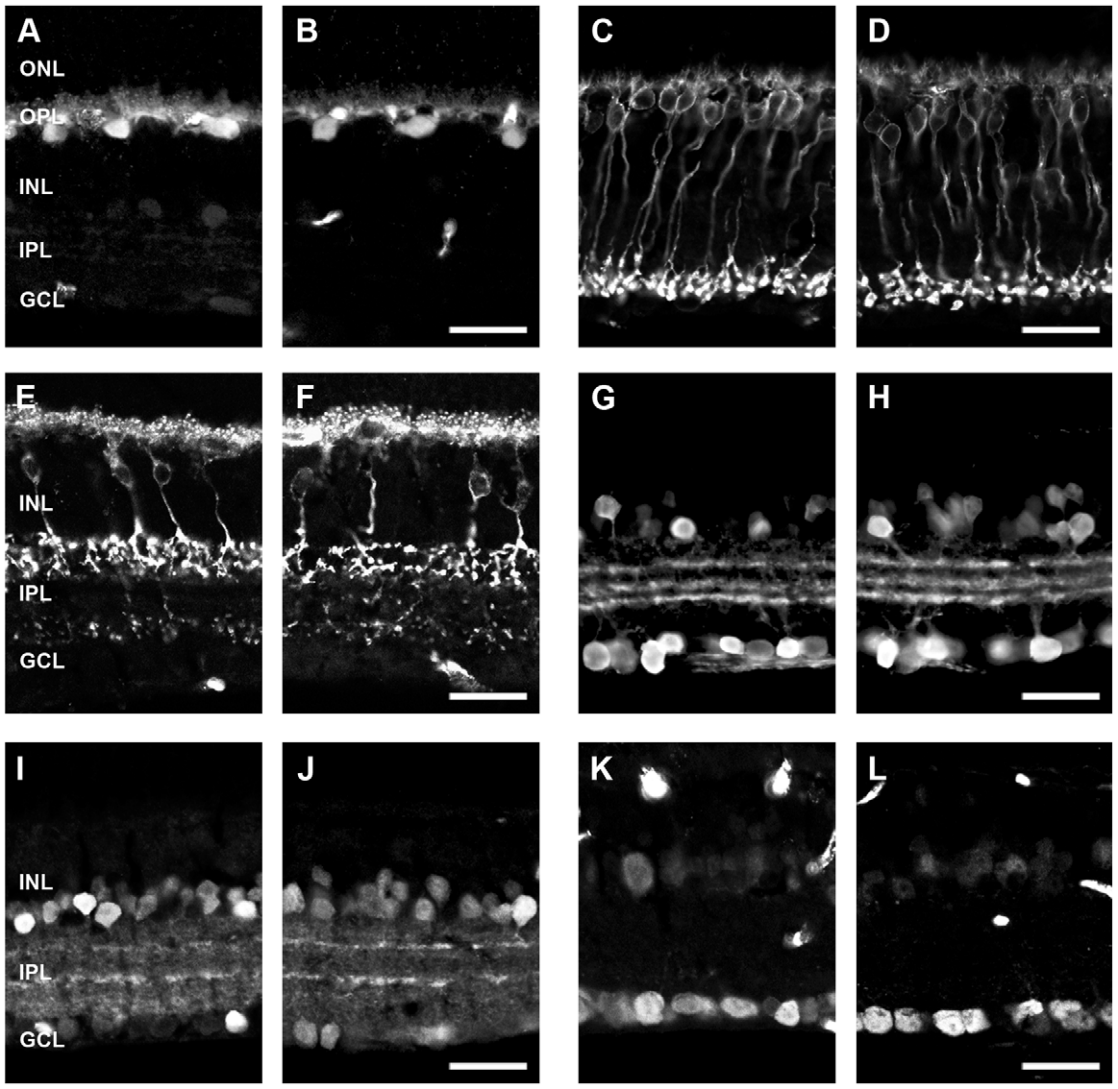
The number and morphology of inner retinal neurons is similar in adult WT and APP-KO mice. Immunohistochemistry using various cell markers was used to probe for gross changes in retinal cell classes in the APP-KO mice. Similar cell profiles were observed for: horizontal cells labelled with Calbindin in (A) WT and (B) APP-KO; rod bipolar cells labelled with PKCα in (C) WT and (D) APP-KO mice; cone bipolar cells (type 2 and 6) labelled with ZNP-1 in (E) WT and (F) APP-KO mice; calretinin-positive amacrine cells in (G) WT and (H) APP-KO mice; GABAergic amacrine cells labelled for GABA in (I) WT and (J) APP-KO mice and; ganglion cells and GABAergic amacrine cells labelled with NueN in (K) WT and (L) APP-KO mice. Outer nuclear layer (ONL), outer plexiform layer (OPL), inner nuclear layer (INL), inner plexiform layer (IPL) and ganglion cell layer (GCL). Scale, 50 microns.

To assess if the differences in ERG b-wave and OP amplitude were due to gross morphological changes in the retina, inner retinal cell markers were used to assess the morphology of the specific cells classes that usually express APP. There were no prominent differences in the labelling profiles of horizontal cells (WT, Figure 6A; APP-KO, Figure 6B), rod bipolar cells (WT, Figure 6C; APP-KO, Figure 6D), type 2-OFF and type 6-ON cone bipolar cells (WT, Figure 6E; APP-KO, Figure 6F), calretinin-positive amacrine cells (WT, Figure 6G; APP-KO retina, Figure 6H), GABAergic amacrine cells (WT, Figure 6I; APP-KO, Figure 6J) and lastly ganglion cells labelled in conjunction with GABAergic amacrine cells using the marker NeuN (WT, Figure 6K; APP-KO, Figure 6L). In our previous study, we showed that INL thickness was subtly but significantly reduced at postnatal day 30 in APP-KO mice [13]. Therefore, further analysis such as quantification of cell or synapse number or morphological analysis of cell structure would be required to identify any subtle changes in the cells that usually express APP, in order to explain the significant functional ERG changes observed between WT and APP-KO mice.

## Discussion

While APP is important in retinal development, its role in modulating retinal function and structure in the adult mouse is unknown. In the current study, we demonstrate that in the adult mouse APP is specifically expressed on neuronal cells in the inner retina and that a lack of APP expression causes significant alterations in the inner retinal function of the rod and cone pathways. However, these changes in retinal function do not translate to a substantial change in visual acuity under light adapted conditions or to changes in the gross cellular structure of some cell classes in the retina. These findings indicate that APP is not required for basic visual function, but rather that it modulates the inner retinal circuitry.

### APP is expressed by neurons of the inner retina of the adult mouse but is not critical for their maintenance

APP expression in the brain is essential for a number of physiological functions [8], [9], [10], [11], [34], [35]. In the eye, APP is expressed in the developing and mature retina and is important for development of retinal cell classes and architecture [2], [13], [36]. To further explore the expression profile of APP in the adult mouse neural retina we used immunohistochemistry with the anti-APP antibody, CT20, to show that APP is expressed on neurons of the inner nuclear layer and ganglion cell layer but not in the outer nuclear layer of the WT mouse. This result is generally consistent with other studies in the retina of the rat [37], human [38] and mouse [13], [36]. In our previous study, APP mRNA was detected in the outer nuclear layer in photoreceptor nuclei using *in situ* hybridisation [13]. In the current study we did not see APP protein in photoreceptor nuclei using immunocytochemistry, however labelling was present in the outer plexiform layer where the protein may be present on the photoreceptor terminals. Moreover, our findings extend on our previous work to show that APP is expressed on horizontal, type 2 cone-OFF bipolar, type 6 cone-ON bipolar, calretinin-positive amacrine, GABAergic amacrine and ganglion cells. The only inner retinal cell types analysed that did not express APP were the rod bipolar cells. This suggests that APP may play a role in the function of specifically horizontal, cone bipolar, amacrine and ganglion cells in the adult WT mouse retina.

Although APP may play a role in the function of these inner retinal neurons, it is not critical for their ongoing survival. Immunohistochemistry for specific retinal cell markers showed that the cell classes that usually express APP were present in both WT and APP-KO retinal tissue and that the morphology of these cells was qualitatively similar between the two genotypes. This suggests that although during early postnatal development there are changes in amacrine cell numbers in APP-KO compared to WT retina, by adulthood, with the exception of the AII amacrine cells, this difference is no longer observed [13]. This may, in part, be explained by the potential for other peptides, such as amyloid beta precursor-like proteins 1 and 2 (APLP1 and APLP2) to compensate for the role of APP during development and in the adult APP-KO mouse [39]. However, studies in the brain of APP-KO mice indicate APP is involved in neurite outgrowth [4] and modulation of synaptic activity [5], [6], [7], [11], as well as being important in synaptic adhesion [12]. If APP plays a similar role in the retina then our findings indicate that APP may be important for synaptic fidelity between inner retinal neurons.

### APP is involved in modulating inner retinal function

The role of APP on the individual components of the rod and cone pathways was assessed using the twin-flash ERG. The rod pathway response of WT and APP-KO mice showed that there was no change in the rod photoreceptor response parameters between the two genotypes. This suggests that there are no significant changes to the number and/or length of the rod outer segments, and no changes in the photoreceptor phototransduction cascade in the APP-KO mice [21], [40]. In contrast, the rod pathway inner retinal responses were significantly different between APP-KO and WT mice. There was a decrease in rod b-wave and an increase in oscillatory potential amplitude in the APP-KO compared to WT mice. Changes in the rod b-wave have been attributed to dysfunction of photoreceptor neurotransmission [41], and dysfunction of rod bipolar cells [24], [25] and/or amacrine cells [42], [43], while changes in oscillatory potential amplitude have been attributed primarily to alterations in amacrine cell responses [22], [42], [43]. Given our immunohistochemical analysis showed that APP is not expressed on rod bipolar cells in the mouse retina, it is unlikely that the changes in the APP-KO rod b-wave are due to alterations of rod bipolar cell function. Rather, it is likely that the rod b-wave is affected by changes in either photoreceptor neurotransmission or alterations in lateral inhibition from the horizontal or amacrine cells. Indeed the rod ERG phenotype observed in APP-KO mice is very similar to that of mice lacking normal lateral inhibition driven by the GABA_C_ receptor [42]. This indicates that the changes in inner retinal function observed in the APP-KO mouse may reflect an alteration in the activity of inhibitory GABAergic amacrine cells, (eg. A17 amacrine cell) which provide feedback to modulate the output of rod bipolar cells [43], [44]. The decrease in cone b-wave amplitude in the APP-KO could be via a similar mechanism to that observed for the rod pathway or via direct alteration to the responses of the cone-bipolar cells themselves [27], [28] given that our findings indicate they usually express APP in the WT mouse.

In contrast to our ERG findings, we found that visual acuity was only subtly different between WT and APP-KO mice across all ages. Considering the substantial changes in the ERG response of the APP-KO mice, an explanation for their relatively normal visual acuity is unclear, but it is likely that the two techniques are assessing the function of different components in the retina. The visual acuity test, under light-adapted conditions, involves the optokinetic eye movement reflex and has been suggested to reflect the direction selective function of the starburst amacrine cells [30], [31], [32], [33], which are unlikely to be stimulated by the stationary flash ERG test. This suggests that APP is not likely to be critical for the development and maintenance of the direction selectivity response of cholinergic/GABAergic starburst amacrine cells. It is possible that APP may only play a role in select amacrine cell pathways within the retina where it might regulate activity by modulating development and synaptic connectivity of certain amacrine classes more so than others. For example, our study and others, implicate a role for APP in modulating the function of amacrine cells in the “through” pathway, eg the GABAergic A17s, which feedback on to rod bipolar cells [42], [44] and the AII amacrine cell [13], while in contrast the function of more complex lateral pathways such as those driven by directionally selective, starburst amacrine cells [45] are less likely to be altered. Our findings indicate that APP is unlikely to be required for basic visual function involved in directional selectivity, but that it may be involved in other modulations of the inner retinal circuitry as indicated by changes in the ERG.

### Implications for Alzheimer's disease and visual function in humans

There is a mounting body of literature correlating Alzheimer's disease with visual disturbances ([46] for review), retinal ganglion cell alterations, in particular nerve fibre layer loss, [47], [48], [49], [50] and also glaucoma [51]. In animal models of glaucoma, the formation of Aβ peptides through cleavage of APP has been reported to be involved in the mechanisms underlying ganglion cell apoptosis [52], [53], [54]. In addition, in animal models of Alzheimer's disease, in which mutant human APP is expressed, the effect of toxic Aβ peptide has been assessed in the retina [2], [36], [55]. These studies found that toxic Aβ peptide was present in the retina [36] and in the ganglion cell layer, and one study found it to be associated with ganglion cell death [2]. Unfortunately, reports confirming the role of APP and Aβ peptides in the normal retina and in the retina of patients with Alzheimer's disease are limited [46]. Our findings with the APP-KO mouse model suggest that APP, or downstream effectors of APP, may be important specifically for inner retina function. Thus, changes in the regulation of retinal APP could contribute to the visual disturbances reported in patients with Alzheimer's disease.

### Conclusion

In conclusion, by utilising a range of functional and histological techniques, we found APP is expressed on inner retinal neurons and that it is required for the inner retinal function of the rod and cone pathway in the adult mouse. However, APP is not required for gross retinal architecture or photopic visual acuity. Our findings indicate that APP is required for normal inner retinal function and provide an insight into the role of APP in the adult retina.
